# Late Arrivals, Early Health Warnings: Post‐Term Birth and Adverse Weight and Metabolic Trajectories

**DOI:** 10.1111/ppe.13154

**Published:** 2025-01-07

**Authors:** Valentina Chiavaroli, Wayne S. Cutfield, José G. B. Derraik

**Affiliations:** ^1^ Neonatal Intensive Care Unit Pescara Public Hospital Pescara Italy; ^2^ Liggins Institute University of Auckland Auckland New Zealand; ^3^ A Better Start, National Science Challenge University of Auckland Auckland New Zealand; ^4^ Department of Paediatrics: Child and Youth Health School of Medicine, Faculty of Medical and Health Sciences, University of Auckland Auckland New Zealand; ^5^ Environmental‐Occupational Health Sciences and Non‐Communicable Diseases Research Group, Research Institute for Health Sciences Chiang Mai University Chiang Mai Thailand; ^6^ Department of Women's and Children's Health Uppsala University Uppsala Sweden

Post‐term birth (≥ 42 0/7 weeks of gestation) affects up to 10% of births globally and, despite advances in obstetric care, is linked to increased risks for both mother and newborn [1, 2, 3]. Evidence suggests that post‐term birth is associated with long‐term adverse health outcomes [3], with compelling evidence of adverse metabolic and adiposity outcomes [4, 5, 6]. Studies have shown that post‐term children exhibit reduced insulin sensitivity and increased body fat compared with their term‐born peers [4], and these risks persist into adolescence [6] and adulthood [5].

However, a recent large study in China involving over 100 thousand children aged 4–7 years reported that post‐term birth was associated with an 18% increased risk of thinness (95% confidence interval [CI], 1.09, 1.28) and a lower risk of overweight/obesity (adjusted relative risk 0.82, 95% CI 0.72, 0.94) [7], contrasting with the existing evidence on this group. In this issue of *Paediatric and Perinatal Epidemiology*, Suhaimi and colleagues provide an additional contribution, using data from the Chinese National Cohort of Motor Development (CNCMD) [8]. The study analysed 141 thousand mother–child pairs, including 7314 (5.2%) children born post‐term who were at higher risk of both thinness and overweight/obesity at ages 3–6 years.

The methodological strengths and limitations of Suhaimi et al.'s study warrant some considerations. The study's retrospective cross‐sectional design and reliance on parental reporting of gestational age introduce potential recall and reporting biases. It also lacks mechanistic detail for the reported outcomes. However, its strengths include its large, representative population sample, comprehensive adjustment for potential confounders and thorough analysis of outcomes across the weight spectrum. In addition, their findings are supported by multiple sensitivity analyses using different classification criteria, including WHO Child Growth Standards, International Obesity Task Force criteria, and Chinese reference data.

Suhaimi et al. report a U‐shaped relationship between gestational age and BMI‐for‐age *z*‐scores, indicating that both preterm and post‐term births were associated with increased BMI‐for‐age *z*‐scores. Their key findings revealed a complex pattern of risk: post‐term children had an increased risk of obesity (+46%), overweight (+27%) and thinness (+13%) compared with those born at term. These associations appeared robust as they were consistently observed across various subgroups.

The paradoxical finding of increased risks for both obesity and thinness suggests complex metabolic programming during prolonged gestation. The authors propose several potential mechanisms to explain this dual pattern [8]. Metabolic disruptions could stem from placental ageing and associated changes in nutrient transport efficiency, and there are also possible hormonal and nutritional imbalances during extended gestation. They note metabolic similarities between post‐term infants and those born small for gestational age, but acknowledge that this pattern is typically observed only at birth. The potential role of genetic factors in individual susceptibility to these outcomes is also considered. While these proposed mechanisms are biologically plausible, the authors rightly emphasise that direct evidence remains limited, particularly regarding the unusual association with thinness, underscoring the need for further research.

The study's subgroup analyses indicated that environmental and clinical factors, including mode of delivery, child's sex, breastfeeding duration and family structure, had minimal influence on modifying these risks. There was limited evidence that thinness and overweight or obesity were less likely to occur among post‐term children from single‐parent families and, to a lesser degree, from extended families. While these findings suggest that familial context may influence weight trajectories in this vulnerable group, the markedly lower number of post‐term children in single‐parent families and the borderline 95% CI for the extended families raise the possibility of statistical artifacts due to reduced sample sizes. Therefore, these observations require replication in other studies.

The timing of adverse outcomes in post‐term children deserves particular attention. Suhaimi et al.'s focus on health outcomes in early childhood provides additional insights, as most of the previous evidence on long‐term associations focused on late childhood, adolescence, or adulthood [4, 5, 6, 9, 10]. In New Zealand, prepubertal children born post‐term (mean age 9.4 years) displayed early markers of metabolic syndrome, notably lower insulin sensitivity and higher central and overall adiposity [4]. Similar findings were noted in a cohort of Swedish boys, where post‐term birth was associated with accelerated weight gain during childhood and increased risk of obesity by age 16 [6]. New Zealand adolescents born post‐term had lower exercise capacity despite similar central cardiac function, which might indicate peripheral vascular adaptations linked to reduced insulin sensitivity [9]. The latter observations were corroborated by a Finnish study of approximately 3500 adults aged 46 years, which reported reduced cardiorespiratory fitness and increased BMI, obesity risk, and insulin resistance in adults born post‐term (*n* = 805), even after adjustment for potential confounding factors [10]. Among 211 thousand 26‐year‐old Swedish women, post‐term birth was also associated with higher adiposity and increased rates of overweight and obesity [5].

This progression of evidence suggests that adverse weight trajectories starting in infancy after post‐term birth may persist and evolve throughout the lifespan and have important implications for clinical practice. The latest identification of both obesity and thinness risks by Suhaimi et al. suggests that post‐term children may benefit from early and regular monitoring of their weight trajectories. The evidence for complex metabolic programming with diverse manifestations in this group indicates that individualised monitoring and intervention may be necessary. Careful management of prolonged pregnancies and targeted research are required to understand and address the mechanisms underlying these outcomes. This is especially the case regarding the reported association between post‐term birth and increased risk of thinness reported in the Chinese studies [7, 8], which, to our knowledge, have been restricted to these two cohorts in China. Therefore, validating these observations in other study populations or long‐term prospective studies is necessary. Such longitudinal studies are essential to fully understand the long‐term implications of post‐term birth and inform interventions that can improve health trajectories for this vulnerable group.

## Conflicts of Interest

The authors declare no conflicts of interest.
